# Post-Modification of Copolymers Obtained by ATRP for an Application in Heterogeneous Asymmetric Salen Catalysis

**DOI:** 10.3390/molecules27144654

**Published:** 2022-07-21

**Authors:** Erigene Bakangura, Philippe Roger, Rafaela S. B. Soares, Mohamed Mellah, Nadine Barroca-Aubry, Anne-Chantal Gouget-Laemmel, François Ozanam, Ludovic Costa, Jean-Pierre Baltaze, Emmanuelle Schulz

**Affiliations:** 1 Centre National de la Recherche Scientifique (CNRS), Institut de Chimie Moléculaire et des Matériaux d’Orsay, UMR 8182, Université Paris-Saclay, 420 Bâtiment, 91405 Orsay, France; bakaeri1@gmail.com (E.B.); rafaela.da-silva-bechara-soares@polytechnique.edu (R.S.B.S.); mohamed.mellah@universite-paris-saclay.fr (M.M.); nadine.aubry-barroca@universite-paris-saclay.fr (N.B.-A.); ludovic.costa@universite-paris-saclay.fr (L.C.); jean-pierre.baltaze@universite-paris-saclay.fr (J.-P.B.); 2 Laboratoire de Physique de la Matière Condensée, Centre National de la Recherche Scientifique (CNRS), Ecole Polytechnique, Institut Polytechnique de Paris, 91120 Palaiseau, France; anne-chantal.gouget@polytechnique.edu (A.-C.G.-L.); francois.ozanam@polytechnique.edu (F.O.)

**Keywords:** copolymerization, ATRP, post-modification, salen, heterogeneous asymmetric catalysis

## Abstract

Copolymers are valuable supports for obtaining heterogeneous catalysts that allow their recycling and therefore substantial savings, particularly in the field of asymmetric catalysis. This contribution reports the use of two comonomers: Azido-3-propylmethacrylate (AZMA) bearing a reactive azide function was associated with 2-methoxyethyl methacrylate (MEMA), used as a spacer, for the ATRP synthesis of copolymers, and then post-functionalized with a propargyl chromium salen complex. The controlled homopolymerization of MEMA by ATRP was firstly described and proved to be more controlled in molar mass than that of AZMA for conversions up to 63%. The ATRP copolymerization of both monomers made it possible to control the molar masses and the composition, with nevertheless a slight increase in the dispersity (from 1.05 to 1.3) when the incorporation ratio of AZMA increased from 10 to 50 mol%. These copolymers were post-functionalized with chromium salen units by click chemistry and their activity was evaluated in the asymmetric ring opening of cyclohexene oxide with trimethylsilyl azide. At an equal catalytic ratio, a significant increase in enantioselectivity was obtained by using the copolymer containing the largest part of salen units, probably allowing, in this case, the more favorable bimetallic activation of both the engaged nucleophile and electrophile. Moreover, the catalytic polymer was recovered by simple filtration and re-engaged in subsequent catalytic runs, up to seven times, without loss of activity or selectivity.

## 1. Introduction

Chiral salen-type metal complexes are known for their catalytic activity, allowing the preparation of scalemic building blocks. Associated with many metals (Cr, Cu, Co, Mn, Fe, etc.), they promote the enantioselective catalytic formation of carbon–carbon or carbon–heteroatom, bonds with excellent efficiency both in terms of activity and selectivity [1,2]. The immobilization of such catalysts is currently particularly under study [3,4,5] to solve two problems; the anchoring of such complexes on a support allows, on the one hand, their easy separation at the end of the reaction, with minimal contamination of the target products in metallic traces. On the other hand, their recovery being greatly facilitated by simple filtration, these robust salen complexes can be engaged again in asymmetric catalytic transformations, leading to an important increase in turnover number (TON). Beyond these two objectives, these immobilization procedures also target the association of these catalytic species in a controlled manner to favor, for example, reactions for which the excellence of the activity and of the enantioselectivity of the process is intimately linked to the efficiency of the cooperativity of two metallic salt centers [6,7,8,9]. In these cases, indeed, the precise location of the active sites is foreseen to favor the activation of both the engaged electrophiles and nucleophiles by two metallic centers, to yield an intermediate possessing the optimized shape in the selectivity-determining transition state for the formation of enantioenriched products [10]. Towards this end, we wanted to exploit the controlled atom transfer radical polymerization (ATRP) process [11,12] implying salen-containing monomers to target uniform chain growth. A styrenyl-modified salen derivative has thus been prepared and engaged in ATRP to aim at controlling the copolymerization of this monomer with styrene, in variable ratios between the two species [13]. Unfortunately, the control of the synthesis could only be obtained when the level of incorporation of the salen comonomer remained below 10%. After complexation with cobalt, these copolymers were able to achieve efficient cooperative activation, leading to the target product in high yields and enantioselectivities, when used as catalysts in the dynamic hydrolytic kinetic resolution of epibromohydrin. Despite its uncontrolled molar mass character, the copolymer containing the highest fraction of the salen unit was shown to be the most effective in terms of enantioselectivity values, in accordance with easier access to cooperative catalysis under these conditions. The resulting catalytic materials were recycled to promote the same reaction, showing stability during their reuse for five cycles.

A different methodology is proposed here to master the control of polymerization with higher active species content for catalysis; the ATRP process is carried out in the presence of suitable monomers. This way, simple analytical techniques demonstrate polymerization control regardless of the proportion of catalytic monomers and the integration of active enantioselective catalytic species by post-modification.

## 2. Results and Discussion

**Scheme 1:**
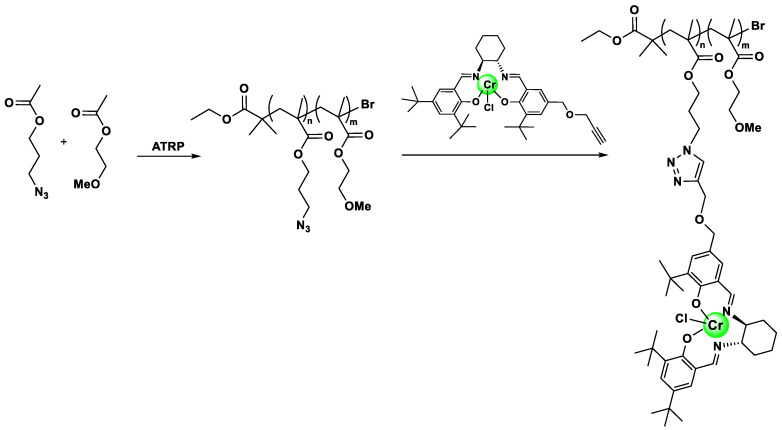
Synthetic route towards chiral chromium salen-containing controlled polymers.

Our goal is to manage the synthesis of a functionalized polymer in a controlled way, which can then be straightforwardly post-functionalized to deliver an active, enantioselective and heterogeneous catalyst to promote asymmetric transformations. The reaction of choice to study the influence of the controlled ATRP on a catalytic transformation is the ring-opening reaction of *meso*-epoxides with trimethylsilylazide [14,15], a reaction for which dual bimetallic activation of each reactive partner is known to be of high importance to reach excellent activity and enantioselectivity. This demanding transformation leads indeed interestingly to precursors of cyclic 1,2-amino alcohols and has been efficiently promoted by chiral chromium salen complexes [16] both in homogeneous and heterogeneous processes [17,18,19,20,21,22]. Such a strategy of using polymer methodologies to target the best composition and location of the catalytic entities has been investigated by the group of Jones for the preparation of polystyrene brushes containing cobalt salen complexes grafted either on silica or on superparamagnetic iron oxide nanoparticles, to evaluate the hydrolytic kinetic resolution of epibromohydrin [23,24]. Nevertheless, the expected controlled character of ATRP could not be definitely achieved. In this context, the proposed synthetic scheme (see Scheme 1) includes two steps: The first one is a copolymerization controlled by ATRP using a functional monomer, azido-3-propylmethacrylate (AZMA), in the presence of a spacer comonomer, 2-methoxyethyl methacrylate (MEMA). In a second step, the chiral chromium salen complex carrying a propargyl function will then be directly incorporated by post-modification by click chemistry using the reactive azide functions of the AZMA units. Post-functionalization on 4-azidomethyl substituted polystyrene by click chemistry with chiral *N*-salicylidene vanadyl(V) *tert*-leucinates has been previously reported by the group of Chen, with the resulting polymeric catalyst promoting the asymmetric aerobic oxidation of α-hydroxy acid derivatives with both high efficiency and recycling ability [25].

### 2.1. ATRP Homopolymerization

The copolymerization of AZMA and MEMA by ATRP was preceded by a kinetic study of the ATRP homopolymerization of each of the two aforementioned comonomers (^1^H NMR shown in Figures S1 and S2 in Supplementary Materials, respectively) The reaction conditions chosen are those used by Sumerlin et al. for the polymerization of AZMA with ethyl 2-bromoisobutyrate (eBiB) as an initiator and anhydrous acetone containing 5 vol% diphenyl ether (DPE) as a solvent at 50 °C with [AZMA]:[eBiB]:[CuBr]:[bpy] = 200:1:1:2 [26].

We have first reproduced a kinetic study of the synthesis of polyAZMA between 1 h and 7 h and the criteria of controlled radical polymerization were indeed verified up to a monomer conversion of 51% (Figures S3 and S4 in Supplementary Materials). A linear increase in -Ln(1-p) (with p being the monomer conversion in %) as a function of time was obtained with an apparent rate constant of 0.11 h^−1^ (Figure S4 in Supplementary Materials). The experimental number-average molar mass M_n_ increased linearly as a function of the conversion p (%). However, as observed in Figure S3 in the Supplementary Materials, these experimental M_n_ values are systematically higher than the theoretical M_n_ data, calculated thanks to the conversion determined by NMR. This can be explained by a possible side chain reaction of the azide function, giving rise to a wider distribution of molar masses. This is corroborated by the dispersity values obtained by SEC between 1.28 and 1.33 (Figure S3 in Supplementary Materials), higher than those obtained by typical ATRP, as previously reported [26].

**Figure 1:**
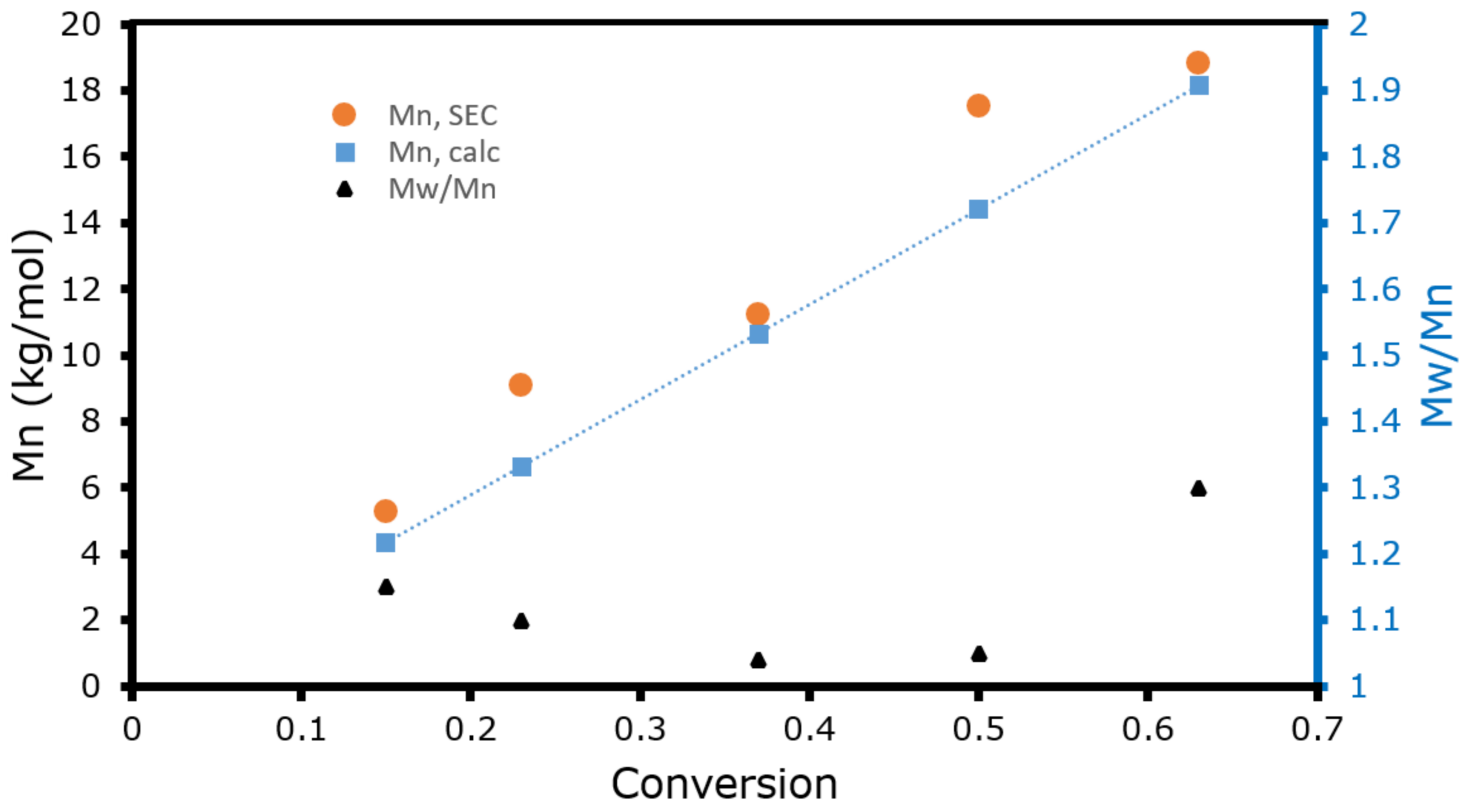
Experimental (Mn SEC) and theoretical (Mn calc) number-average molecular mass, and dispersity versus conversion for polyMEMA.

**Figure 2:**
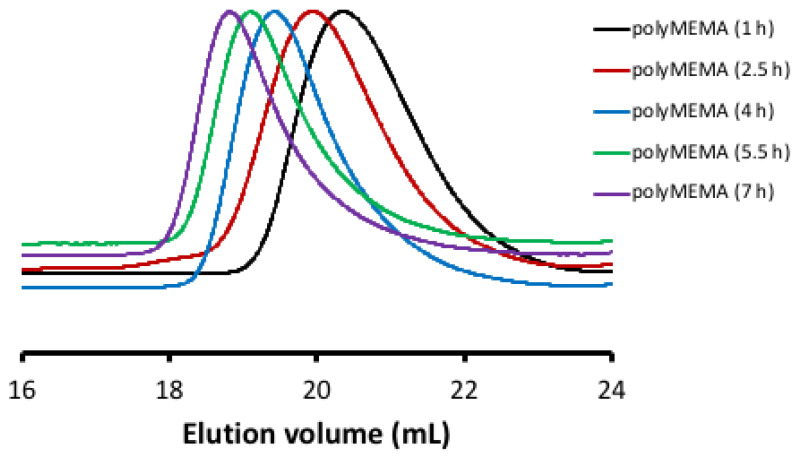
SEC profiles of polyMEMA.

A kinetic study of the ATRP synthesis of polyMEMA was then realized under the same conditions as those used for polyAZMA. To the best of our knowledge, the kinetics of the controlled homopolymerization of MEMA has never been reported up to now, despite the frequent use of this monomer in copolymerization reactions [27,28]. The kinetic results indicate that the polymerization satisfies all the criteria of a controlled polymerization in molar mass for polymerization times ranging from 1 h to 7 h, up to a monomer conversion of 63%. The semi-logarithmic kinetic representation indicates an increase in Ln [M]_0_/[M] = −Ln(1−p) as a function of time with an apparent rate constant of 0.13 h^−1^ (Figure S4 in Supplementary Materials). Absolute number-average molar masses M_n_ obtained by SEC-MALS are close to the theoretical M_n_ calculated thanks to the conversion determined by NMR (see Figure 1). As expected, the number-average molar mass M_n_ obtained by SEC linearly increases as a function of the conversion p (%). Finally, the SEC profiles (Figure 2) show monomodal and narrow molar mass distributions with dispersities ᴆ (M_w_/M_n_) varying between 1.04 and 1.14, characteristic of a controlled polymerization in molar mass for a conversion up to 63%.

Figure S4 in the Supplementary Materials represents the semilogarithmic kinetic plots for the ATRP homopolymerizations of MEMA and AZMA with [monomer]:[eBiB]:[CuBr]:[bpy] = 200:1:1:2, in anhydrous acetone (with 5 vol% DPE) at 50 °C. This figure demonstrates that the apparent rate constants are very close (K_app_ = 0.13 h^−1^ and 0.11 h^−1^). This indicates that both comonomers should be incorporated at the same rate during the copolymerization reaction, considering that the reactivity ratios of AZMA and MEMA are similar. This must actually be the case given the fact that these two monomers have an identical chemical structure at the level of the polymerizable methacrylate function.

### 2.2. ATRP Copolymerization

Considering these data, three copolymerizations were performed during 6 h under the same experimental conditions as before, with initial comonomer loadings MEMA/AZMA (mol%) of 90/10, 70/30 and 50/50, targeting approximately the same final comonomer incorporation in the copolymers. The obtained polymers are designated as Copo1, Copo2 and Copo3 in the remainder of the text, respectively.

**Figure 3:**
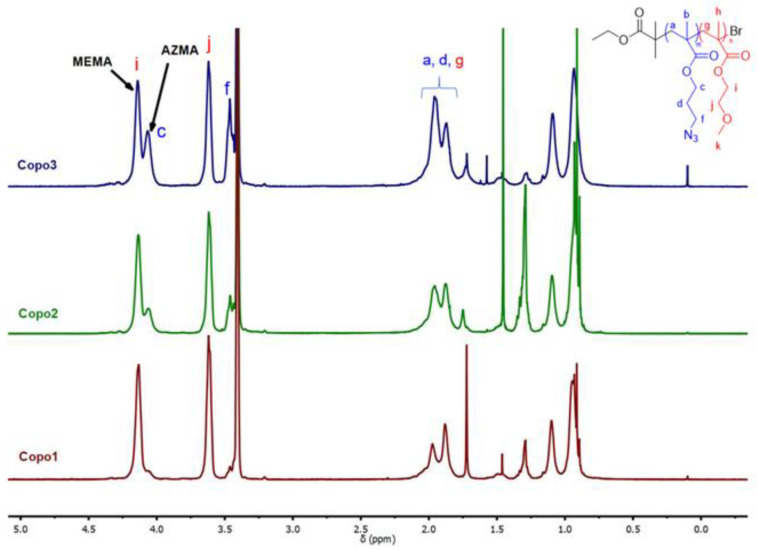
NMR spectra of copolymers.

NMR spectra (Figure 3) clearly demonstrate the difference in the compositions of the three copolymers. The complete assignment of the signals corresponding to the protons of each comonomer is to be found in Figure S5 in the Supplementary Materials. Thus, the calculation of the ratio of the intensities of the two signals corresponding to the chemical shifts of the protons belonging, respectively, to AZMA (δ_Hc_ = 4.02 ppm) and to MEMA (δ_Hi_ = 4.10 ppm) leads to a percentage of AZMA incorporation of 6, 24 and 53%, close to the percentage of the comonomer feed composition, which was 10, 30 and 50%, respectively. The signals located around 2 ppm correspond to the two methylene groups of each monomer in the backbone (Ha and Hg for AZMA and MEMA, respectively), together with the methylene (Hd) in the substituent of the AZMA monomer. The calculation made from the evolution of their intensities leads to similar results.

As can be observed in Table 1, the composition of the copolymers obtained by NMR is also in agreement with the initial comonomer feed, with values of 7, 22 and 40%, respectively. There is an increase of 8, 11 and 20% in the incorporation of MEMA compared to its initial feed. This greater incorporation of MEMA relative to AZMA in the copolymer increases with conversion.

**Figure 4:**
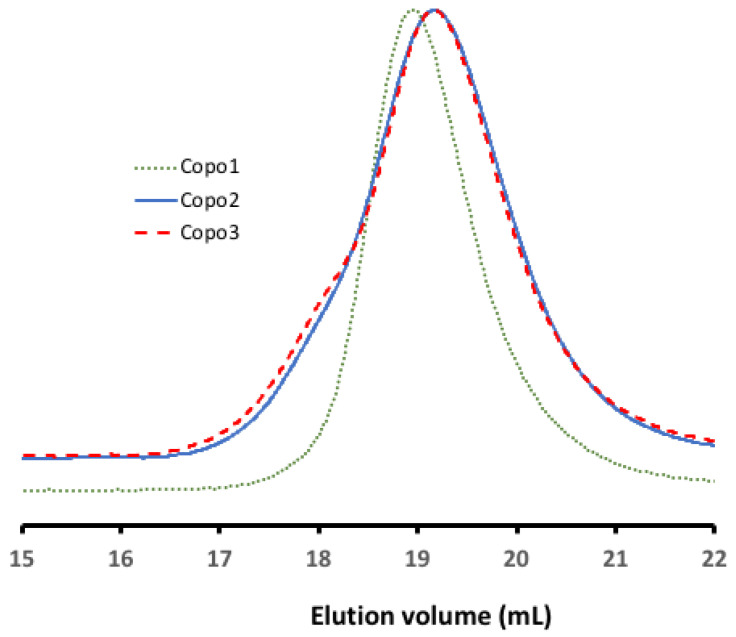
Evolution of SEC profiles of copolymers.

This observation is validated by the kinetic results obtained for the two homopolymers (see Figure S4 in the Supplementary Materials). The increase in dispersity ᴆ from 1.05 to 1.28 is correlated with the composition of AZMA. The copolymerization reaction is indeed less controlled in molar mass when the initial percentage of AZMA is higher, which is consistent with the homopolymerization results. The broader molar mass distributions of Copo2 and Copo3 are compared to the narrower molar mass distributions of Copo1, as observed by the SEC profiles in Figure 4. The SEC profiles of all copolymers were unimodal, assessing the efficiency of the copolymerization versus homopolymerization. The discrepancy between the calculated number-average molar masses and the number-average molar masses obtained by SEC for Copo2 and Copo3 in Table 1 can also be explained by the higher dispersity values obtained for higher AZMA content.

### 2.3. DOSY NMR Experiments

DOSY experiments were also performed to provide further evidence for the efficiency of the copolymerization. In this type of experiment, a single translational diffusion coefficient is obtained if the copolymerization is quantitative. Otherwise, several diffusion coefficients would be found. For example, if two homopolymers are synthesized, then two diffusion coefficients should be observed by DOSY NMR.

**Figure 5:**
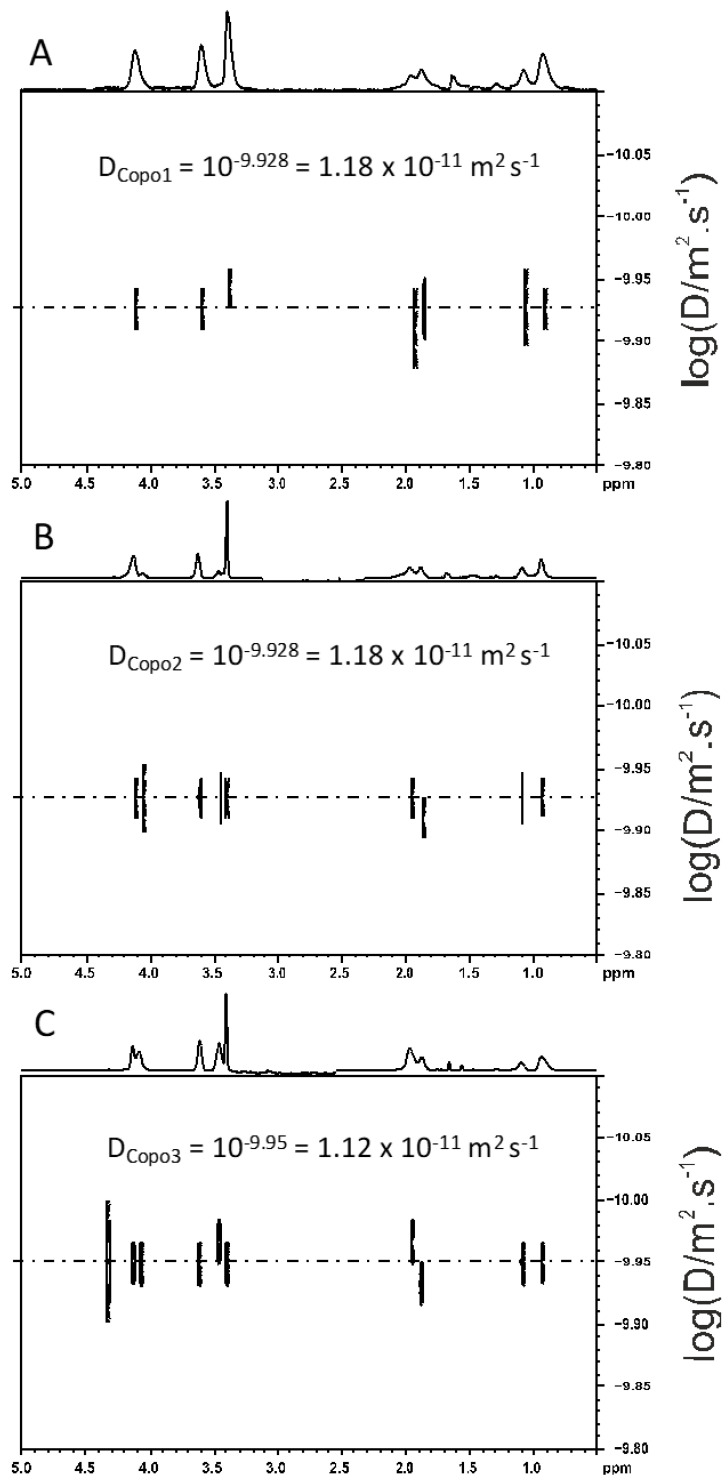
DOSY NMR of copolymers: (**A**) Copo1, (**B**) Copo2 and (**C**) Copo3.

Figure 5 shows, for each copolymer, that all the NMR signals are correlated to a single value of the translational diffusion coefficient D. The values of D are identical for Copo1 and Copo2: D = 1.18 × 10^−10^ m^2^s^−1^. For Copo3, the value of D decreased down to D = 1.12 × 10^−10^ m^2^s^−1^. These values are not significantly different (considering a precision of 10% on the value of D). This result is not surprising given that the diffusion coefficient depends on the molar mass of the polymer: D ~ M^−ν^ (with ν being a scaling law coefficient) and that, in our case, all three copolymers are expected to have a very similar molar mass (see Mn calc, Table 1).

### 2.4. IR Characterization

**Figure 6:**
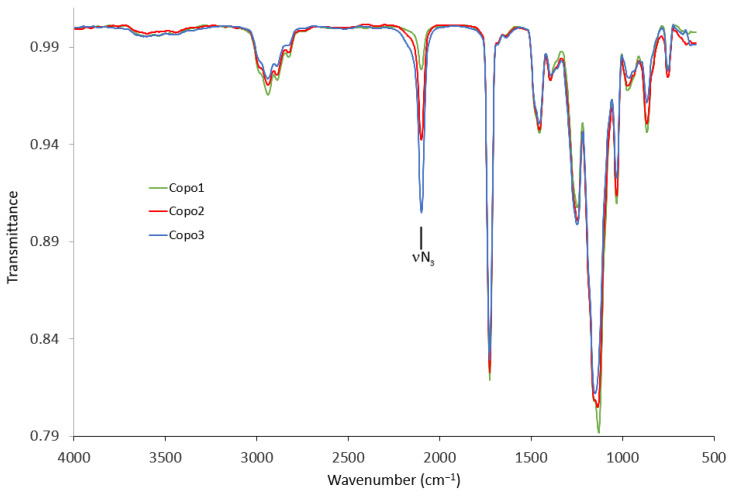
ATR-IR spectra of copolymers.

The chemical composition of the synthesized copolymers was also assessed by FTIR spectroscopy in the range of 4000 to 600 cm^−1^ (Figure 6). Mainly, the bands at 2950 and 2900 cm^−1^ were associated with the stretching vibration modes of the CH_2_ from aliphatic moieties. A strong azide stretching mode is observed at 2090 cm^−1^, and the C = O stretching mode is observed at 1725 cm^−1^ and C-O at 1130 cm^−1^. The intensity of the azide band is clearly increasing with the AZMA content in the copolymers. To determine the AZMA content, the ratio of the transmittance of the azide band to the transmittance of the C = O band at 1725 cm^−1^ can be considered because this last band is present in all methacrylate units (neglecting the C = O contribution of the initiator). Therefore, the ratio of the peak intensities I(N_3_)/I(CO) leads to a percentage of AZMA of 13:32:55, almost similar to the AZMA feed at 10:30:50.

### 2.5. Preparation of the Chromium Salen Copolymer Catalysts

**Scheme 2:**
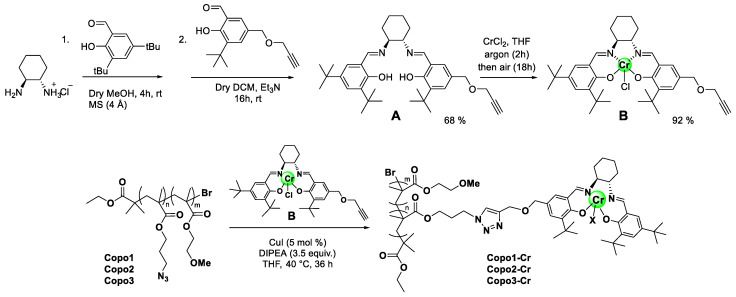
Chromium salen complex synthesis and click reactions with Copo1–3.

With these three azide-functionalized copolymers in hand, a “clickable” enantioselective chromium catalyst was thus synthesized, following a two-step procedure. The procedure described by Weck et al. was reproduced to afford the enantiopure unsymmetrical salen ligand (A) [29]. This implies the prior preparation of the (1*S*,2*S*)-cyclohexane-1,2-diamine monoammonium salt [29] and of the 3-*tert*-butyl-2-hydroxy-5-((prop-2-ynyloxy)methyl)benzaldehyde [25]. The novel targeted salen derivative was then isolated in 68% yield (see Scheme 2). Insertion of chromium in the coordinating sites was finally performed from CrCl_2_ in THF under an argon atmosphere, followed by oxidation in air [30].

**Figure 7:**
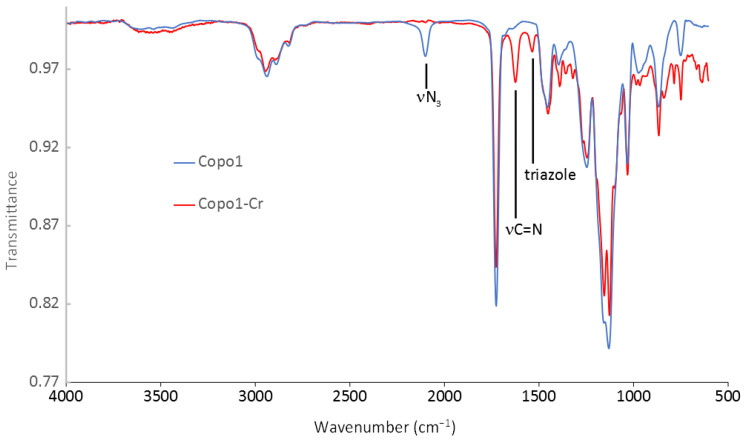
ATR-IR spectra of Copo1 (in blue) and Copo1-Cr (in red).

Subsequent azide–alkyne Huisgen cycloadditions promoted by Cu (I) salts were conducted to react the propargyl-tagged chromium salen complex with the azide groups of all three copolymers [22,25], delivering in high yield the corresponding supported salen catalysts, as brown powders. Completion of this reaction was verified by FTIR spectroscopy, since the typical strong azide band at 2090 cm^−1^ fully disappeared, at least for Copo1 and Copo2, after 36 h of stirring at 40 °C (see Figure 7 as illustration for Copo1, and Figures S6 and S7 for Copo2 and Copo3 in Supplementary Materials). A residual band at 2090 cm^−1^ is nevertheless still present for Copo3, indicating an incomplete Cr–salen addition. However, in each case, a peak at 1538 cm^−1^ appears clearly, characteristic of a triazole-cycle deformation mode [31]. A new peak at 1622 cm^−1^ is, moreover, visible, which can be attributed to the υC = N stretching vibration of the salen complex [14]. The three supported catalysts were then recovered and thoroughly washed with an aqueous ethylenediamine tetraacetic acid solution to remove residual copper salts, dried and further used as catalysts to promote the ring opening of cyclohexene oxide with trimethylsilylazide.

### 2.6. Asymmetric Heterogeneous Catalysis

**Scheme 3:**
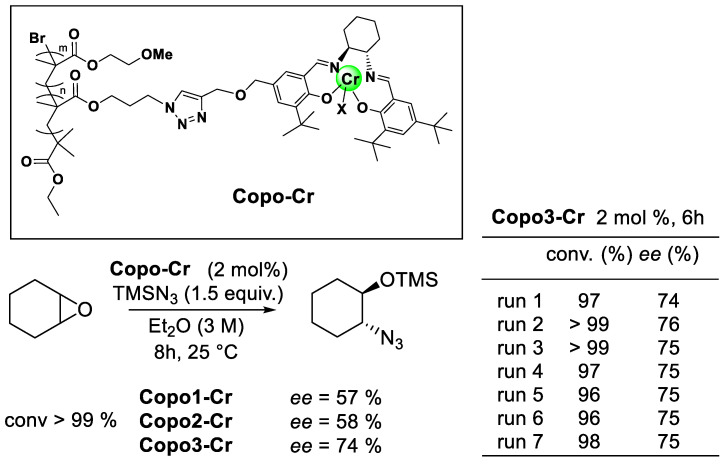
Asymmetric ring opening of cyclohexene oxide in the presence of Copo-Cr catalysts.

This catalytic reaction was performed in the presence of the supported catalysts containing 2 mol% of active sites relative to the engaged substrate, in diethyl ether, a solvent in which the polymeric complexes are almost insoluble. When performed in our hands with the classical Jacobsen chromium catalyst, the ring-opened product was obtained with 88% *ee*, but full conversion was not achieved after 24 h of reaction. This was further confirmed during the preparation of a racemic sample of ((2-azidocyclohexyl)oxy)trimethylsilane with a racemic Jacobsen chromium catalyst, which only led to a conversion of 27% after 8 h of reaction. On the contrary, all attempts involving the use of the three Copo-Cr polymer complexes reached full conversion in only 8 h of reaction, under the same dilution conditions, indicating an efficient accelerating effect that is not usual in heterogeneous catalysis (Scheme 3 and Figure S8 for conversion determination by GC analysis in Supplementary Materials) [32].

This high activity was accompanied by variations in terms of enantioselectivity values, according to the composition of the catalyst (see Figures S9 and S10 in Supplementary Materials for the determination of the enantiomeric excesses). Indeed, the copolymers for which the catalyst was diluted by the presence of the MEMA units (Copo1-Cr and Copo2-Cr) delivered the ring-opened product in 57–58% *ee*. Conversely, Copo3-Cr, which is supposed to contain an equivalent amount of MEMA units and catalytically active sites, afforded the product with 74% *ee*. We assume that this higher enantioselectivity is due to the enhanced possibility of performing a bimetallic activation, with two Cr-active sites placed in proximity to activate both the epoxide and the nucleophile.

The recyclability of this catalyst, giving the best activity and selectivity values, was then explored. As all the Copo-Cr species were insoluble in the reaction solvent, they were easily filtered from the reaction mixture, to be engaged in a next run with renewed addition of both substrates. This procedure was, in particular, tested in the presence of Copo3-Cr, for which the supernatant solution was recovered after a first run, carried out at room temperature for 6 h of reaction time, sufficient to obtain complete conversion. The catalyst was washed with diethyl ether at 0 °C, dried and reused in subsequent additional six runs (see table in Scheme 3). The second use of this recovered supported catalyst was also very efficient, as were subsequent ones, allowing seven uses of the same batch of catalyst. Delightfully, both the activity of the catalyst and the enantioselectivity values of the recovered product remained stable, at the exact level of the first cycle, i.e., a total conversion and an enantiomeric excess stable at 75%.

## 3. Materials and Methods

### 3.1. General Information

Firstly, 3-*tert*-butyl-2-hydroxylbenzaldehyde, trioxane, (1*S*,2*S*)-1,2-cylohexanediamine, HCl 1M in diethyl ether, tetrabutylammonium iodide (TBAI) and sodium hydride (NaH) were purchased from Aldrich (Saint-Louis, MO, USA) and used as received. Moreover, 3-(*tert*-butyl)-5-(chloromethyl)-2-hydroxylbenzaldehyde [13] and (1*S*,2*S*)-1,2-cyclohexanediamine mono (hydrogen chloride) [29] were synthesized as described in previously published work. Dichloromethane, methanol, ethanol and diethyl ether were dried before use. Copper bromide was purified by treating it with glacial acetic acid and washed several times with ethanol absolute and diethyl ether. Diphenyl ether was dried on molecular sieves. The AZMA synthesis was performed according to a described procedure [26].

^1^H and ^13^C NMR were recorded on a Bruker AV360 spectrometer (Billerica, MA, USA), operating at 360 MHz for ^1^H NMR, and 90 MHz for ^13^C NMR. Chemical shifts are reported downfield from CDCl_3_ (δ: 7.26 ppm, for ^1^H NMR and δ: 77.0 ppm for ^13^C NMR), used as an internal reference. Data are represented as follows: chemical shift, multiplicity (br = broad, s = singlet, d = doublet, t = triplet, q = quartet, m = multiplet), coupling constants in Hertz (Hz), integration. ESI-HRMS was detected on a Bruker MicroTOF-Q daltonics spectrometer (Billerica, MA, USA) by electrospray ionization (ESI). GC analyses were performed on a Shimadzu GC 2010 (Kyoto, Japan) plus using a chiraldex β-PM column (50 m × 0.25 mm × 0.12 µm) and hydrogen as a carrier gas (isothermal 110 °C). Conversion was calculated using dodecane as an internal standard. Attenuated Total Reflectance (ATR) FTIR was carried out on fine powder of the studied copolymers. In essence, the copolymer powder was deposited on the diamond crystal of an ATR module from Pike Technologies (Fitchburg, WI, USA) and the infrared spectrum was recorded using a Bruker IFS 66 spectrometer (Billerica, MA, USA). Two hundred scans of resolution 4 cm^−1^ were recorded between 600 cm^−1^ and 4000 cm^−1^. Spectra visualization and treatment were done using OPUS software (Toronto, ON, Canada); spectra integration was done using the built-in integration function of Origin^®^ v8.0724. Size Exclusion Chromatography (SEC) analysis of polymers was carried out at 35 °C, using THF as an eluent. Typically, the polymer solution was prepared at 4 mgmL^−1^ and then filtered through a 0.45 μm PTFE filter to remove insoluble residues. The separation system included one guard column (Malvern TGuard, Malvern, UK) and two separation columns: (1) Viscotek LC3000L (Malvern, UK) (300 × 8.0 mm) and (2) ViscoGEL^TM^ GM_HH_ r-H (300 × 7.8 mm, Malvern, UK). The intensities were recorded using a refractive index (RI) detector (Walter 410; Tübingen, Germany) and a multi-angle light scattering (MALS) detector (Viscotek SEC-MALS 20, Malvern, UK). Absolute number and weight-average molar masses were calculated using OmniSec™ 5.12.467 software, distributed by Malvern Panalytical (Malvern, UK). The refractive index increment (dn/dC) of 0.078 mLg^−1^, determined by Stejskal et al. for polyMEMA in THF at 546 nm, was also used for polyAZMA based on their roughly similar chemical structures [33].

### 3.2. Method for the Synthesis of the Alkyne-Modified Salen Ligand and the Corresponding Chromium Complex

#### 3.2.1. Synthesis of 3-tert-butyl-2-hydroxy-5-((prop-2-ynyloxy)methyl) Benzaldehyde

In a three-necked bottom flask, sodium hydride (60% dispersion in mineral oil, 1.5 eq, 7.96 mmol, 0.316 g) was suspended in 15 mL of dry THF under an argon atmosphere. The solution was cooled down at 0 °C in an ice bath. Propargyl alcohol (1.5 eq, 7.96 mmol, 463 µL) in 15 mL of dry THF was added dropwise, and the mixture was stirred at ambient temperature for 4 h. The reaction mixture was cooled at 0 °C and then 3-(*tert*-butyl)-5-(chloromethyl)-2-hydroxybenzaldehyde (1 eq, 5.30 mmol, 1.2 g) in 15 mL of dry THF was added dropwise and TBAI (0.03 eq, 0.16 mmol, 0.058 g) was added under an argon atmosphere. The reaction mixture was allowed to stir while refluxing for 16 h and was then cooled down at 0 °C. Deionized water (2 mL) was slowly added. Solvents were removed under reduced pressure. The obtained residues were partitioned in dichloromethane (30 mL) and deionized water (30 mL). The aqueous layer was extracted two times by DCM (30 mL). The organic phase was combined, washed with brine solution (2 × 60 mL) and dried over MgSO_4_. After filtration and removal of solvents under reduced pressure, the crude product was purified by column chromatography (hexane/AcOEt; 19/1). The product was obtained as a brown viscous liquid (320 mg, 24% yield). ^1^H NMR (360 MHz, CDCl_3_): δ 11.80 (s, 1H), 9.87 (s, 1H), 7.51 (d, 1H, *J* = 2.0 Hz), 7.41 (d, 1H, *J* = 2.0 Hz), 4.56 (s, 2H), 4.20 (s, 2H), 2.49 (s, 1H), 1.42 (s, 9H), see Figure S11 in Supplementary Materials. These data are identical to those previously described [25].

#### 3.2.2. Synthesis of the Salen Ligand Bearing a Propargyl Moiety

First, (1*S*,2*S*)-1,2-cyclohexanediamine mono(hydrogen chloride) (1 eq, 1 mmol, 150.6 mg), 3,5-di-*tert*-butyl-2-hydroxylbenzaldehyde (1 eq, 1 mmol, 234.3 mg) and a molecular sieve (4 Å, 0.83 g) were charged into a Schlenk tube. The tube was then evacuated three times and filled with argon. Under the argon atmosphere, 9 mL of anhydrous methanol was added, and the mixture was stirred at room temperature for 4 h. A solution of 3-*tert*-butyl-2-hydroxy-5-((prop-2-ynyloxy) methyl)benzaldehyde (1.1 eq, 1.1 mmol, 273 mg) in 9 mL of dried dichloromethane and 0.42 mL of anhydrous triethylamine (3 eq, 3 mmol) was added to the above mixture and was stirred at room temperature for 18 h. The reaction mixture was filtered through a short pad of celite and flushed with anhydrous dichloromethane. After removal of the solvent under reduced pressure, the crude product was purified by column chromatography on silica gel (hexane/AcOEt/Et_3_N: 95/5/1) to afford the title product as a yellow powder (382 mg, 68% yield). ^1^H NMR (360 MHz, CDCl_3_): δ 14.02 (s, 1H), 13.69 (s, 1H), 8.31 (s, 1H), 8.30 (s, 1H), 7.33 (d, *J* = 2.5 Hz, 1H), 7.25 (d, *J* = 2.1 Hz, 1H), 7.03 (d, *J* = 2.1 Hz, 1H), 6.99 (d, *J* = 2.5 Hz, 1H), 4.47 (s, 2H), 4.11 (d, *J* = 2.4 Hz, 2H), 3.34 (m, 2H), 2.45 (t, *J* = 2.4 Hz, 1H), 2.00–1.87 (m, 4H), 1.81–1.71 (m, 2H), 1.55–1.46 (m, 2H), 1.44 (s, 9H), 1.43 (s, 9H), 1.25 (s, 9H), see Figure S12 in Supplementary Materials. ^13^C NMR (90 MHz, CDCl_3_): δ 166.01, 165.38, 160.44, 158.05, 140.03, 137.44, 136.46, 130.14, 129.93, 126.95, 126.10, 126.07, 118.40, 117.86, 79.89, 74.63, 72.56, 72.48, 71.56, 56.76, 35.08, 34.89, 34.14, 33.28, 31.54, 29.55, 29.43, 24.44, see Figure S13 in Supplementary Materials. FTIR (cm^−1^): 3299; 2952; 2865; 2093; 1627; 1448; 1360; 1263; 1206; 1168; 1087; 1036; 979; 936; 870; 776; 708; 652. HRMS (M + H^+^): calc for C_36_H_51_N_2_O_3_; 559.3894 found for C_36_H_51_N_2_O_3_: 559.3864.

#### 3.2.3. Synthesis of the Chromium Salen Complex Bearing a Propargyl Moiety

A solution of the salen ligand (1eq, 0.358 mmol, 200 mg) in dry, degassed THF (5 mL) was assessed with a solution of anhydrous CrCl_2_ (1,15 eq, 0.538 mmol, 66 mg) in dry, degassed THF (8 mL). The resulting brown solution was stirred under argon for 2 h and then in air for an additional 18 h. The solution was then diluted with dichloromethane and washed with saturated NH_4_Cl and brine. The organic phase was dried over anhydrous MgSO_4_ and filtered. The solvent was removed under reduced pressure to afford the salen complex as a brown powder (213 mg, 92% yield). FTIR (cm^−1^): 3306, 2950, 2868, 2093, 1622, 1538, 1440, 1392, 1350, 1319, 1250, 1203, 1166, 1078, 1031, 971, 924, 871, 828, 787, 742, 665. HRMS (M-Cl): calc for C_36_H_48_CrN_2_O_3_; 608.3070; found for C_36_H_48_CrN_2_O_3_ 608.3029.

### 3.3. General Procedure for the Homopolymerization: Poly (MEMA) Synthesis

A mixture of dry acetone (3 mL) and diphenyl ether (DPE) (0.2 mL) was deoxygenated in a Schlenk tube by 5 freeze–pump–thaw cycles. CuBr (0.005 eq, 0.15 mmol, 21.4 mg) and 2,2′-bipyridine (bpy) (0.01 eq, 0.30 mmol, 46.8 mg) were added under an argon atmosphere. Vacuum was applied, followed by 3 freeze–pump–thaw cycles. Concomitantly, 2-methoxyethyl methacrylate (MEMA) was passed through an Al_2_O_3_ column to remove inhibitors and then degassed by argon gas bubbling, followed by 3 freeze–pump–thaw cycles. To the mixture containing acetone/DPE/CuBr/bpy, MEMA (1 eq, 30.00 mmol, 4.3 g) was added using inert syringe techniques. Two cycles of vacuum and argon flow were applied and the mixture was placed in a preheated oil bath at 50 °C. While stirring, ethyl 2-bromoisobutyrate (eBiB) (0.005 eq, 0.125 mmol, 18 μL) was added. The mixture was allowed to stir for 6 h and, periodically, samples were taken in between for the kinetics study. After completion of the reaction, the mixture was dissolved in DCM and filtered through a short column of Al_2_O_3_. The solvent was then removed under reduced pressure and the polymer was precipitated in cold methanol under stirring. For the NMR studies, the sample taken from the reaction was immediately solubilized in CDCl_3_ and then filtered in a small pipette plugged with cotton and filled with Al_2_O_3_ to remove the CuBr (Figure S2 in Supplementary Materials shows the NMR of PolyMEMA synthesis at partial conversion at T5). The following proton NMR description only describes the signals for the polymer: ^1^H NMR (360 MHz, CDCl_3_) δ 4.09 (s, 2H), 3.58 (s, 2H), 3.36 (s, 3H), 1.84 (s, 2H), 1.09 and 0.91 (2 bs, 3H). For poly(AZMA) synthesis, see Supplementary Materials.

### 3.4. General Procedure for the Copolymerization

A mixture of dry acetone (3 mL) and DPE (0.5 mL) was degassed in a Schlenk tube by 5 freeze–pump–thaw cycles. Concomitantly, MEMA was passed through an Al_2_O_3_ column to remove inhibitors. Then, a mixture of MEMA (see quantity indicated below) and 3-azidopropyl methacrylate (AZMA) (see quantity indicated below) was degassed in a Schlenk tube by 3 freeze–pump–thaw cycles. Over the acetone/DPE mixture, CuBr (see quantity indicated below) and bpy (see quantity indicated below) were added under an argon atmosphere. The MEMA and AZMA mixture was subsequently added using inert syringe techniques. Three new freeze–pump–thaw cycles were applied to the mixture, and then it was placed in a preheated oil bath at 50 °C. While stirring, eBiB (see quantity indicated below) was added. The mixture was allowed to stir for 6 h and, periodically, samples were taken in between for the kinetics study.

**Copo1**; MEMA90-co-AZMA10

2-methoxyethyl methacrylate (0.90 eq, 20.00 mmol, 2.88 g), 3-azidopropyl methacrylate (0.10 eq, 2.20 mmol, 0.37 g), CuBr (0.005 eq, 0.11 mmol, 0.016 g), bpy (0.01 eq, 0.22 mmol, 0.035 g), and eBiB (0.005 eq, 0.11 mmol, 15.9 μL). Isolated yield 87%.

**Copo2**; MEMA70-co-AZMA30

2-methoxyethyl methacrylate (0.7 eq, 19.00 mmol, 2.73 g), 3-azidopropyl methacrylate (0.30 eq, 8.10 mmol, 1.37 g), CuBr (0.005 eq, 0.135 mmol, 0.019 g), bpy (0.01 eq, 0.27 mmol, 0.042 g), and eBiB (0.005 eq, 0.135 mmol, 19.6 μL). Isolated yield 86%.

**Copo3**; MEMA50-co-AZMA50

2-methoxyethyl methacrylate (0.5 eq, 11 mmol, 1.58 g), 3-azidopropyl methacrylate (0.5 eq, 11 mmol, 1.86 g), CuBr (0.005 eq, 0.11 mmol, 0.016 g), bpy (0.01 eq, 0.22 mmol, 0.035 g), and eBiB (0.005 eq, 0.11 mmol, 15.9 μL). Isolated yield 83%.

### 3.5. General Procedure for the Post-Modification by Azide–Alkyne Huisgen Cycloaddition

Poly(MEMAn-co-AZMAm) and Salen B were dissolved in 5 mL of degassed dry THF in a Schlenk tube. This mixture was then degassed by 3 freeze–pump–thaw cycles. Over this frozen mixture during the last cycle, CuI was added and allowed to dissolve under vacuum. *N*,*N*-diisopropylethylamine (DIPEA) was added under an argon atmosphere. The mixture was placed in a preheated oil bath at 40 °C for 36 h, under constant stirring. Then, 30 mL of dry THF was added to the mixture, and it was filtered through Al_2_O_3_ column chromatography. The solvent was removed under reduced pressure and the product was precipitated in excess of hexane. The product was filtered, washed with hexane and dried under vacuum. The method for calculating the quantity of each of the species to be introduced is detailed in the Supplementary Materials, for obtaining Copo1-Cr, Copo2-Cr and Copo3-Cr.

### 3.6. Catalytic Procedure (ARO Reaction) in Heterogeneous Conditions

A tube was charged with the considered Copox-Cr (2 mol%) and maintained under argon. Diethyl ether (300 µL), cyclohexene oxide (0.305 mmol) and dodecane as an internal standard (20 µL) were then added. The reaction mixture was stirred for 10 min at 25 °C before adding the trimethylsilyl azide (60 µL, 0.458 mmol), and then stirred for 6 h. A sample was prepared to calculate conversion by GC (and *ee*). The supported catalyst was washed with diethyl ether (15 mL) at 0 °C, filtered and dried in the tube for its reuse. (([1*R*,2*R*]-2-azidocyclohexyl)oxy)trimethylsilane; ^1^H NMR (360 MHz, CDCl_3_) δ (ppm): 3.46–3.40 (m, 1H), 3.22–3.14 (m, 1H), 1.95–1.84 (m, 2H), 1.70–1.60 (m, 2H), 1.41–1.12 (m, 4H), 0.16 (s, 9H). These analytical data are in accordance with reported ones [34]. The *ee* was determined by chiral GC (isothermal 110 °C), which resolved both enantiomers (tr_maj_= 10.90 min, tr_min_= 11.29 min). The absolute stereochemistry was assigned as (1*R*,2*R*) based on comparison with the literature [14]. The method for calculating the quantity of Copox-Cr to be introduced is detailed in the Supplementary Materials.

## 4. Conclusions

In summary, we have described the controlled copolymerization of AZMA and MEMA by ATRP and demonstrated the efficiency of this procedure up to an equimolar mixture of the two monomers. A preliminary rigorous study of the monomers’ homopolymerization has indeed indicated that the kinetics of the transformation of both species remained similar for the control of the molar mass and the composition. The obtained copolymers were carefully characterized thanks to ^1^H NMR and SEC analyses. NMR DOSY experiments further confirmed the efficiency of the procedure since unique and close values for the diffusion coefficients were obtained in each case. FTIR analyses before and after the click reaction of the azide functionalities with the alkyne-substituted chiral chromium salen complex firstly confirmed the AZMA content in each copolymer and secondly demonstrated the quantitative formation of the triazole ring, at least for the two copolymers with the lowest AZMA ratios. Under the same experimental conditions, the copolymer with the highest AZMA content led to an incomplete Cr–salen addition. Each copolymer was engaged as a heterogeneous catalyst to promote the asymmetric aminolysis of cyclohexene oxide with TMSN_3_ at a low catalyst loading (2 mol%) and provided the targeted open product in almost quantitative yield at room temperature, regardless of its composition. These heterogeneous catalysts appear more active than their homogeneous analogues, even if the enantioselectivity values remain lower. Nevertheless, the highest selectivity of the series (74% *ee*) was reached by using Copo3, in which the active site proportion is the largest, conditions for which bimetallic catalysis is most favorable. As an insoluble species, Copo3 was also easily recovered by filtration and recommitted to catalysis after solvent washing, drying and further addition of substrates. This procedure could be repeated seven times to produce the target compound with particularly stable values in terms of activity and enantioselectivities. The post-modification of copolymers obtained by ATRP by chiral organometallic complexes therefore makes it possible to obtain high-value insoluble asymmetric catalysts, with the control of the composition paving the way to the preparation of tailor-made polymetallic catalysts for applications in the innovative field of asymmetric heterogeneous multicatalysis.

## Figures and Tables

**Table 1:** Copolymerization of MEMA and AZMA in anhydrous acetone at 50 °C with 5 vol% DPE with [M]:[eBiB]:[CuBr]:[bpy] = 200:1:1:2 for a polymerization time of 6 h.

	Comonomer Feed MEMA/AZMA (%/%)	Conversions ^a^ p_MEMA_/p_AZMA_ (%/%)	Mn _calc_ ^b^ (g/mol)	Mn_SEC_^c^ (g/mol)	ᴆ ^c^	Composition ^d^ MEMA/AZMA (%/%)
Copo1	90/10	73/71	21,300	22,500	1.05	93/7
Copo2	70/30	64/61	19,200	25,700	1.20	78/22
Copo3	50/50	66/63	21,200	30,800	1.28	60/40

^a^ Obtained from ^1^ H-NMR (see Supplementary Materials, Figure S1 (P_AZMA_) and Figure S2 (P_MEMA_)). ^b^ Calculated from NMR conversions: Mn _calc_ = M_EtBriBu_ + p_MEMA_ × M_0 MEMA_ × DP_target(MEMA)_ + p_AZMA_ × M_0 AZMA_ × DP_target(AZMA)_. ^c^ Obtained from SEC analysis. ^d^ Obtained from NMR analysis of the copolymers (see Supplementary Materials, Figure S5).

## Data Availability

Data are contained within the article and Supplementary Materials.

## Supplementary Materials

Synthesis and catalysis details, kinetics results, copolymer characterization, NMR spectra and GC chromatograms are available online at https://www.mdpi.com/article/10.3390/molecules27144654/s1, Figure S1: ^1^H NMR of PolyAZMA_T6; Figure S2: ^1^H NMR of PolyMEMA_T5; Figure S3: Experimental Mn (Mn SEC), theoretical Mn (Mn calc) and dispersity versus conversion for polyAZMA with [monomer]:[eBIB]:[CuBr]:[bpy]; 200:1:1:2 in anhydrous acetone (with 5 vol% DPE) at 50 °C; Figure S4: Semilogarithmic kinetic plot of MEMA and AZMA with [monomer]:[eBIB]:[CuBr]:[bpy]; 200:1:1:2 in anhydrous acetone (with 5 vol% DPE) at 50 °C; Figure S5: ^1^H NMR of Copo2 after 6 h reaction; Figure S6: ATR-IR spectra of Copo2 (in blue) and Copo2-Cr (in red); Figure S7: ATR-IR spectra of Copo3 (in blue) and Copo3-Cr (in red); Figure S8: GC spectrum of cyclohexenoxide (Ret.Time 3.22 min) using dodecane (Ret.Time 5.81 min) as a standard; Figure S9: GC spectrum of a racemic sample of ((2-azidocyclohexyl)oxy)trimethylsilane; Figure S10: GC spectrum of a 75% ee sample of ((2-azidocyclohexyl)oxy)trimethylsilane; Figure S11: ^1^H NMR of 3-tert-butyl-2-hydroxy-5-((prop-2-ynyloxy)methyl)benzaldehyde; Figure S12: ^1^H NMR of salen ligand with propargyl moiety; Figure S13: ^13^C-NMR spectrum of salen ligand with propargyl moiety; Table S1: ATRP of MEMA with [MEMA]:[eBiB]:[CuBr]:[bpy]; 200:1:1:2 in 40 vol% anhydrous acetone and 2.6 vol% diphenyl ether at 50 °C.

## Sample Availability

The samples are not available from the authors.
