# Supplementary material for: Post-Modification of Copolymers Obtained by ATRP for an Application in Heterogeneous Asymmetric Salen Catalysis
Source: Molecules. 2022 Jul 21;27(14):4654. doi: 10.3390/molecules27144654 (PMC9319095; doi:10.3390/molecules27144654)
Supplement: Supplementary file 1 [file molecules-27-04654-s001.zip › molecules-1814656-supplementary.pdf]

SUPPORTING INFORMATION

Table of Contents

I. Homopolymerization

I.1. Poly(AZMA) synthesis

I.2. Poly(MEMA) synthesis

I.3. Kinetic results

II. Copolymerization

III. Post-modification of the copolymers by azide-alkyne Huisgen cycloaddition

IV. Catalytic procedure (ARO reaction) in heterogeneous conditions

V. GC traces for the ARO reaction in heterogeneous conditions

VI. ^{1}H and ^{13}C NMR spectra

I. Homopolymerization

I.1. Poly(AZMA) synthesis

A mixture of dry acetone (3 mL), DPE (0.5 mL) and 3-azidopropyl methacrylate (1 eq, 17 mmol, 2.87 g) was deoxygenated in a Schlenk tube by 5 freeze–pump–thaw cycles. CuBr (0.005 eq, 0.085 mmol, 12 mg) and bipyridine (0.01 eq, 0.17 mmol, 0.027 g) were added under an argon atmosphere. The Schlenk was sealed with a septum and 3 cycles of argon backfilling were applied. The mixture was placed to a preheated oil bath at 50 °C; then, while stirring, ethyl-2-bromoisobutyrate (eBiB) (0.005 eq, 0.085 mmol, 12.3 µL) was added. The mixture was allowed to stir for 7 hours and, periodically, samples were taken in between for the kinetics study. After completion of the reaction, the mixture was dissolved in DCM and filtered through a short column of Al_2O_3. The solvent was then removed under reduced pressure and the polymer was precipitated in cold methanol under stirring. For the NMR studies, the sample taken from the reaction was immediately solubilized in $CDCl_3$ and then filtered in a small pipette plugged with cotton and filled with Al_2O_3 to remove the CuBr (Figure S1 shows the NMR of PolyAZMA synthesis at partial conversion at T6). The following proton NMR description only describes the signals for the polymer ^1H NMR (360 MHz, $CDCl_3$ 4.03 (bs, 2H), 3.39 (bs, 2H), 1.98 – 1.88 (m, 2H), 1.03 and 0.87 (2 bs, 3H)).

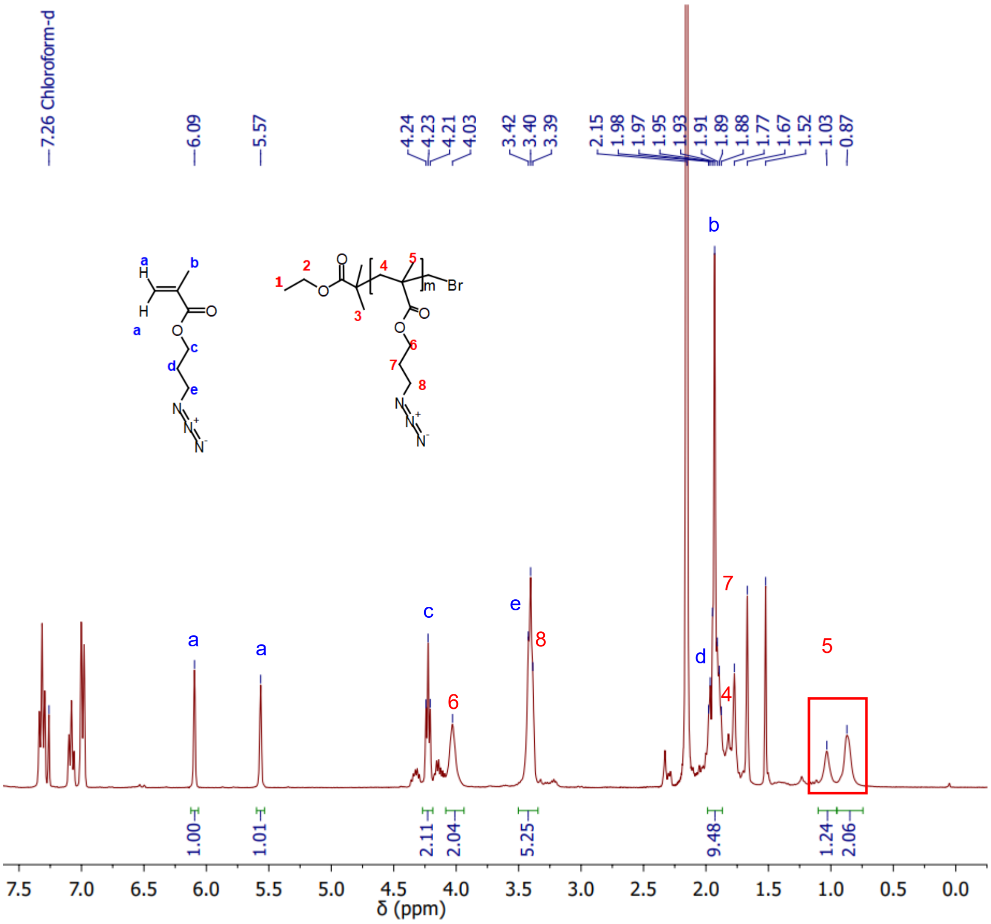

Figure S1: ^{1}H NMR of **PolyAZMA_T6.** Peaks from 6.91 to 7.38 ppm are attributed to bipyridine. Peak at 2.16 ppm refers to acetone.

The NMR conversion was calculated by dividing the integral of H_6 (from the polymer) by the sum of H_a (from the monomer) and H_6. For example,

$$p_{AZMA} = \frac{H_{6\,(polymer)}}{H_{a\,(monomer)} + H_{6\,(polymer)}} = \frac{2.04}{(2 + 2.04)} = 0.51$$

I.2. Poly(MEMA) synthesis

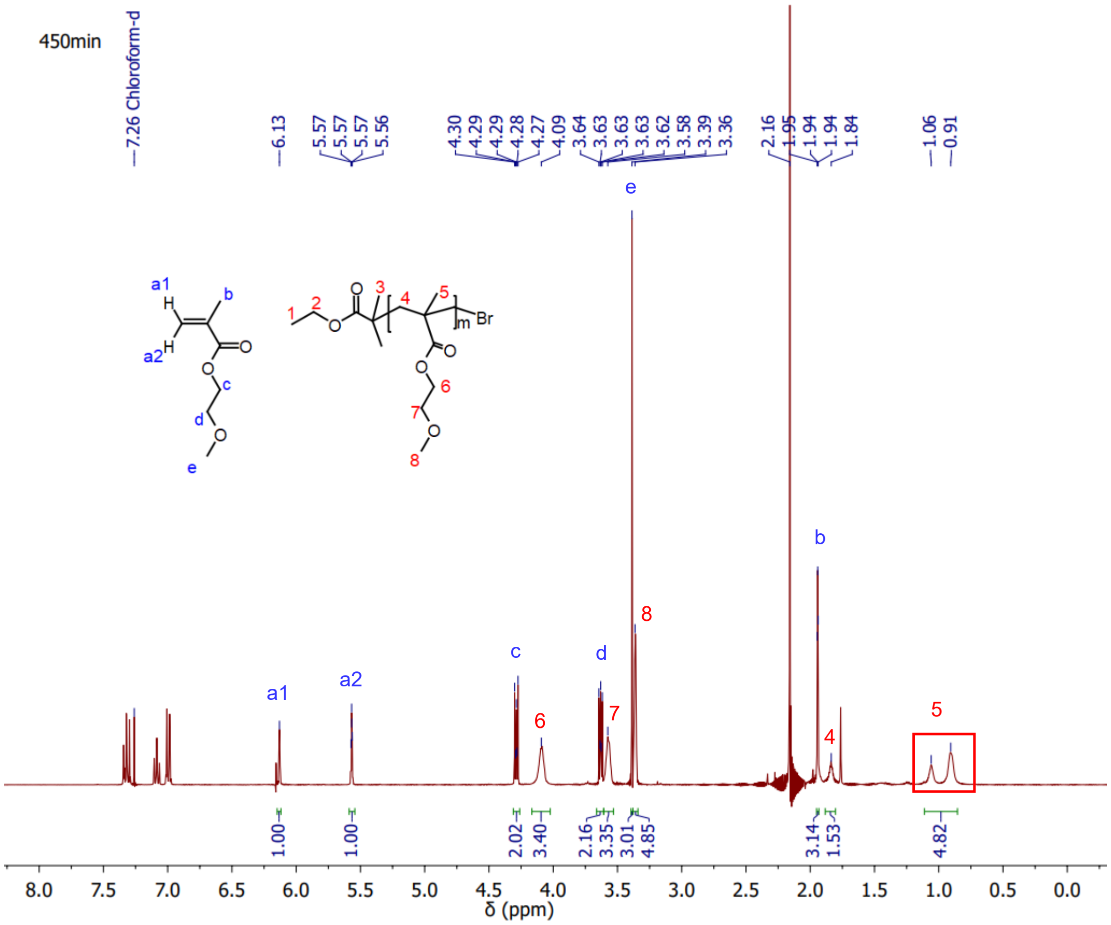

Figure S2: ^{1}H NMR of **PolyMEMA_T5.** Peaks from 6.91 to 7.38 ppm are attributed to bipyridine. Peak at 2.16 ppm refers to acetone.

The NMR conversion was calculated by dividing the integral of H_6 (from the polymer) by the sum of H_a (from the monomer) and H_6. For example,

$$p_{MEMA} = \frac{H_{6\,(polymer)}}{H_{a\,(monomer)} + H_{6\,(polymer)}} = \frac{3.4}{(2 + 3.4)} = 0.63$$

I.3. Kinetics results

Figure S3 shows experimental Mn (Mn SEC), theoretical Mn (Mn calc) and dispersity versus conversion for polyAZMA.

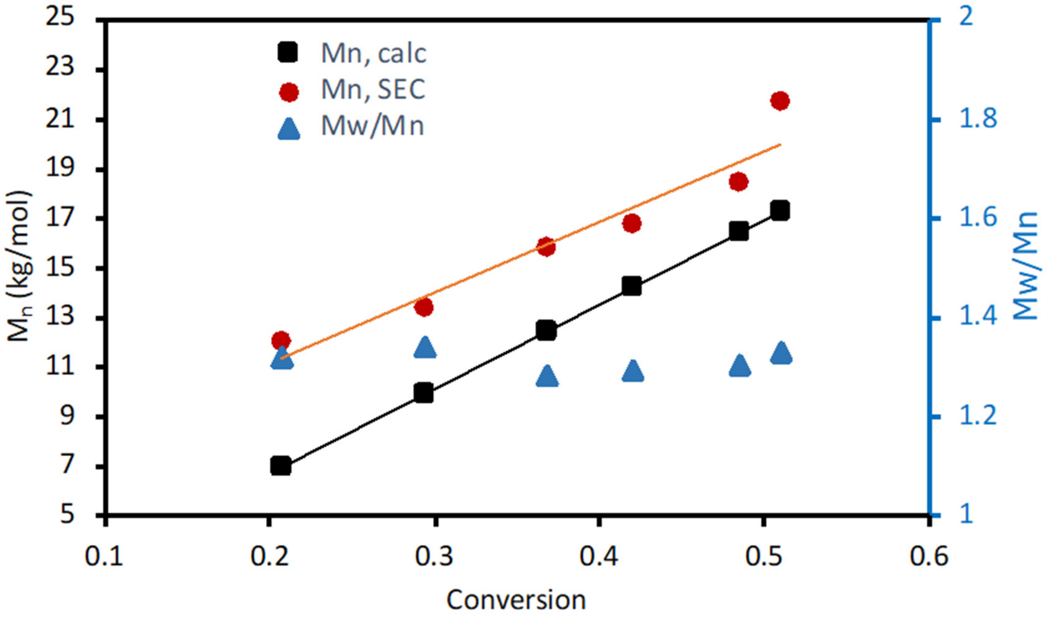

Figure S3: Experimental Mn (Mn SEC), theoretical Mn (Mn calc) and dispersity versus conversion for polyAZMA with [monomer]:[eBIB]:[CuBr]:[bpy]; 200:1:1:2 in anhydrous acetone (with 5 vol% DPE) at 50 °C.

Table S1 shows the values regarding the kinetics of MEMA followed by NMR and SEC analysis.

Table S1: ATRP of MEMA with [MEMA]:[eBiB]:[CuBr]:[bpy]; 200:1:1:2 in 40 vol % anhydrous acetone and 2.6 vol% diphenyl ether at 50 °C.

	Time (h)	Conversion (%) NMR	Mn cal (g/mol) [a]	Mn,SEC (g/mol)[b]	**Đ**[c]
**PolyMEMA_T1**	1	15	4400	5300	1.05
**PolyMEMA_T2**	2.5	23	6700	9100	1.10
**PolyMEMA_T3**	4	37	10600	11200	1.04
**PolyMEMA_T4**	5.5	50	14400	17500	1.05
**PolyMEMA_T5**	7	63	18100	18800	1.14

[a] Calculated from ^{1}H-NMR; [b] obtained from SEC analysis; [c] dispersity (Mw/Mn)

The semilogarithmic kinetic plot of the ATRP of both MEMA and AZMA is shown in Figure S4.

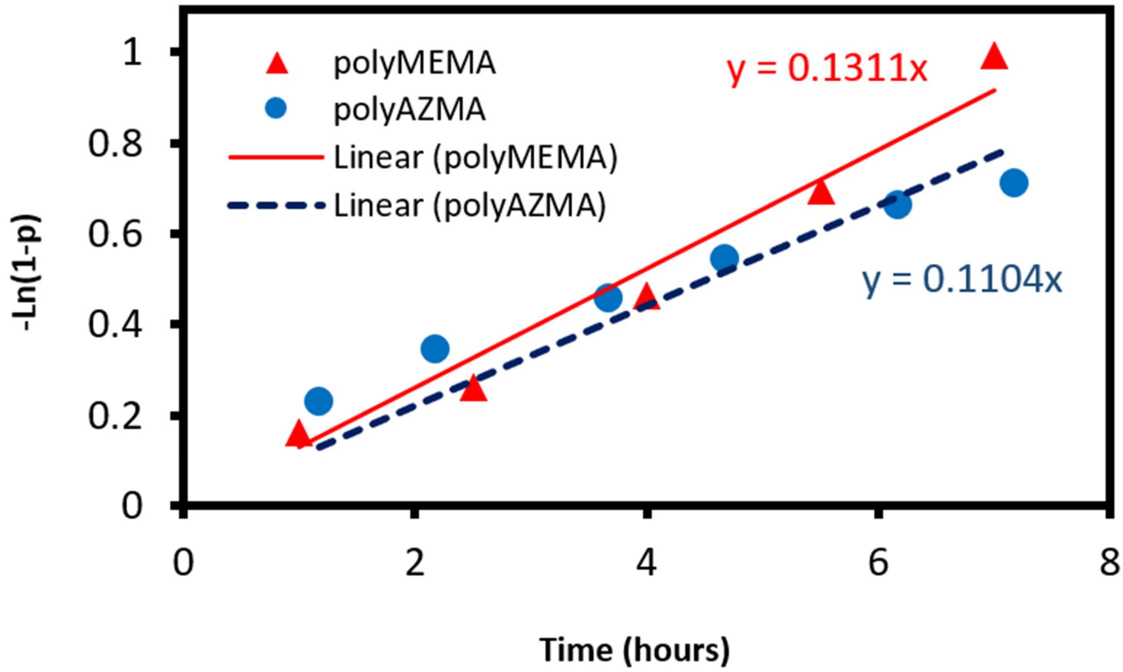

Figure S4: Semilogarithmic kinetic plot of MEMA and AZMA with [monomer]:[eBIB]:[CuBr]:[bpy]; 200:1:1:2 in anhydrous acetone (with 5 vol% DPE) at 50 °C. p is the monomer conversion.

II. Copolymerization

The ^{1}H-NMR spectra of poly(MEMAn-co-AZMAm) were key for the calculation of the percentage composition of MEMA and AZMA units in the copolymers. Figure S5 shows the NMR of the copolymerization of MEMA and AZMA at a 70/30 molar ratio after 6 hours of reaction. The NMR conversions can provide the actual molar ratio of the polymerization by using the formulas below.

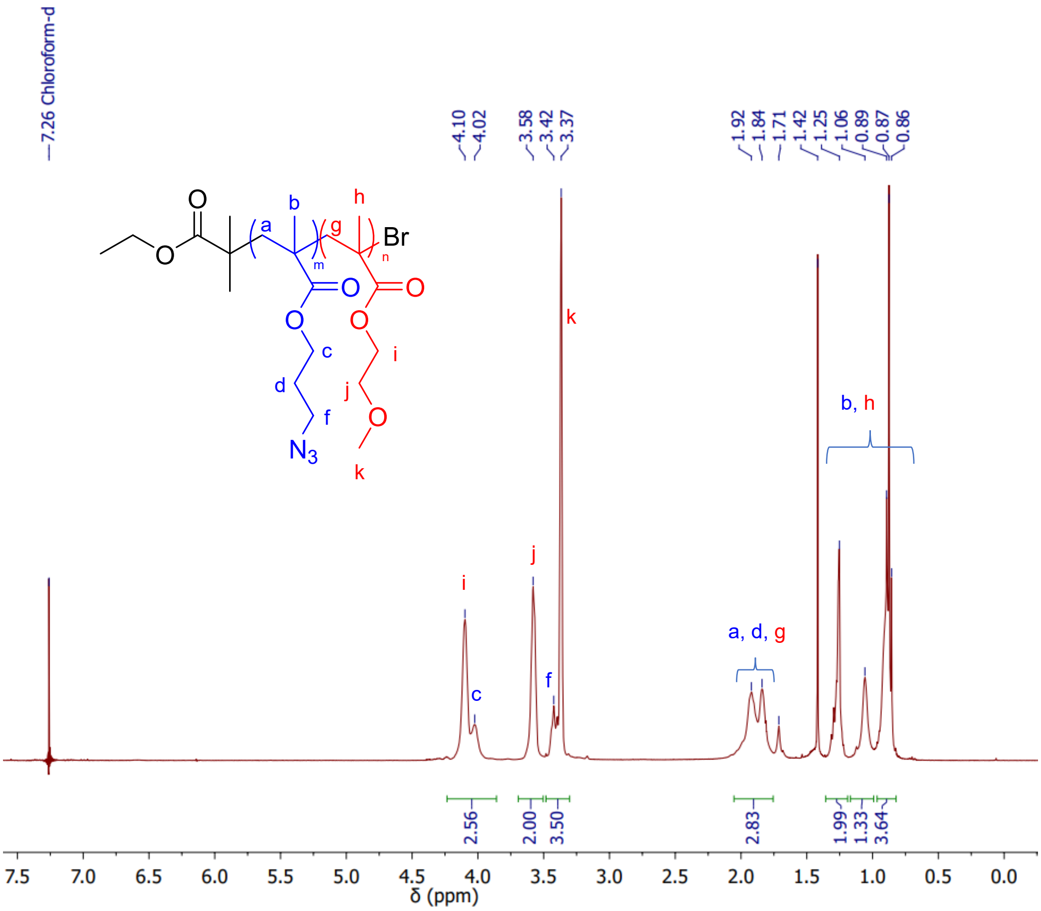

Figure S5: ^{1}H NMR of **Copo2** after 6 h reaction.

MEMA (%) $= \frac{H_{(\delta=3.58\ ppm)}\ x\ 100}{(H_{(\delta=4.10\ ppm)}+H_{(\delta=4.02ppm)})} = \frac{2\ x\ 100}{(2+0.56)} =$ 78%	AZMA (%) $= \frac{H_{(\delta=4.02\ ppm)}\ x\ 100}{(H_{(\delta=4.10\ ppm)}+H_{(\delta=4.02ppm)})} = \frac{0.56\ x\ 100}{(2+0.56)} =$ 22%

III. Post-modification of the copolymers by azide-alkyne Huisgen cycloaddition

The amounts of copox and salen B involved in the click reaction were calculated according to the following method.

The molecular weight of **Copox** (Mw_{Copox}) was calculated according to the NMR data, namely DP_{AZMA} states for the degree of polymerization of AZMA monomer; the salen unit is introduced in a 1.5-fold excess compared to the azido unit contained in the copolymers.

$$n_{salen} = (1.5 * DP_{AZMA} * m_{Copox}) / Mw_{Copox}$$

Thus, for

Copo1: $\mathbf{Mw}_{\mathbf{Copo1}}$ = 21300 g.mol^{-1}, with 71 % conversion of AZMA, and DP_{AZMA} = 14;

Copo2: $\mathbf{Mw}_{\mathbf{Copo2}}$ = 19200 g.mol^{-1}, with 61 % conversion of AZMA, and DP_{AZMA} = 37;

Copo3: $\mathbf{Mw}_{\mathbf{Copo3}}$ = 21200 g.mol^{-1}, with 63 % conversion of AZMA, and DP_{AZMA} = 63.

Preparation of Copo1-Cr; **Copo1** (1 eq, 0.07 mmol, 100 mg), Salen **B** (1.5 eq, 0.1 mmol, 64.4 mg), CuI (10 %, 1.3 mg), DIPEA (3.5 eq, 0.245 mmol, 31.7 mg).

Preparation of Copo2-Cr; **Copo2** (1 eq, 0.16 mmol, 80 mg), Salen **B** (1.5 eq, 0.23 mmol, 148.8 mg), CuI (10%, 2.9 mg), DIPEA (3.5 eq, 0.53 mmol, 69.5 mg).

Preparation of Copo3-Cr; **Copo3** (1 eq, 0.13 mmol, 44 mg), Salen B (1.5 eq, 0.19 mmol, 126 mg), CuI (10%, 2.5 mg), DIPEA (3.5 eq, 0.47 mmol, 60.8 mg).

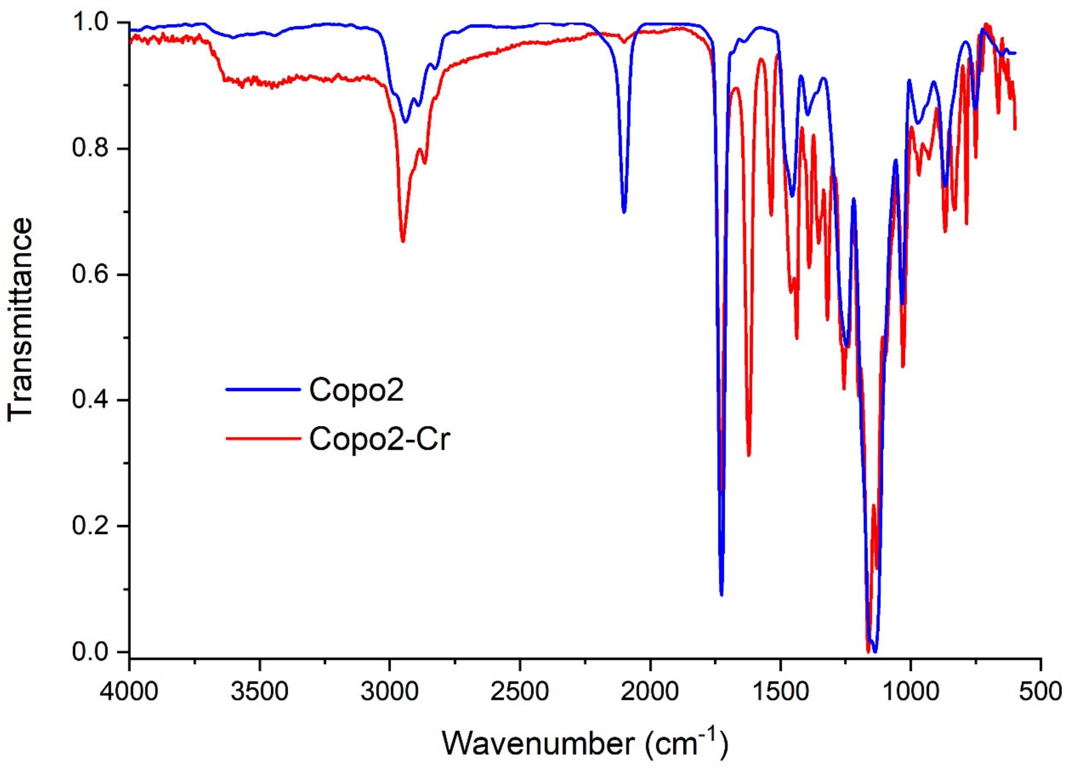

Figure S6: ATR-IR spectra of **Copo2** (in blue) and **Copo2-Cr** (in red).

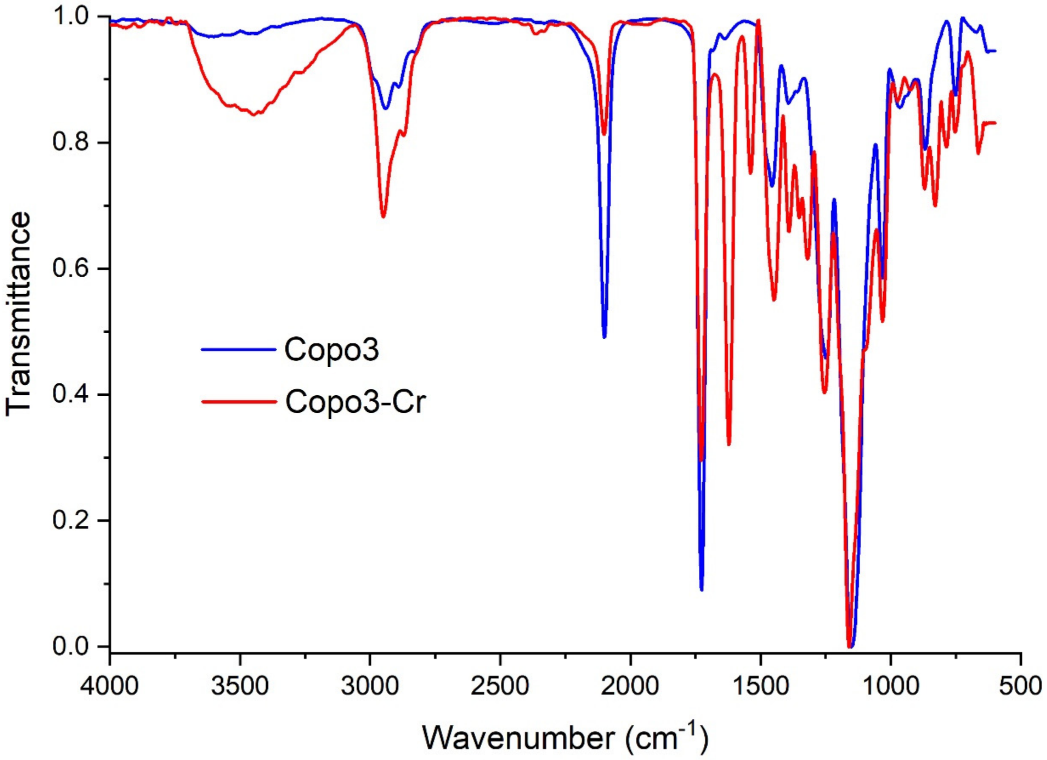

Figure S7: ATR-IR spectra of **Copo3** (in blue) and **Copo3-Cr** (in red).

IV. Catalytic procedure (ARO reaction) in heterogeneous conditions

The amount of weighted **Copox-Cr** ($m_{Copox\text{-}Cr}$) to be introduced in the reaction mixture was calculated as follows. The molecular weight of **Copox-Cr** ($Mw_{Copox\text{-}Cr}$) was calculated according to the NMR data, considering the conversion of each monomer and the molecular weight of MEMA and of the monomer unit resulting from the click reaction of the salen-Cr complex with AZMA, and DP_{AZMA} states for the degree of polymerization of AZMA monomer.

$$m_{Copox\text{-}Cr} = (0.02 \times n_{epoxide} \times Mw_{Copox\text{-}Cr}) / DP_{AZMA}$$

Thus, for

Copo-1-Cr: $Mw_{Copo1\text{-}Cr}$ = 31037 $gmol^{-1}$, with 71 % conversion of AZMA, and DP_{AZMA} = 14;
Copo-2-Cr: $Mw_{Copo2\text{-}Cr}$ = 45131 $gmol^{-1}$, with 61 % conversion of AZMA, and DP_{AZMA} = 37;
Copo-3-Cr: $Mw_{Copo3\text{-}Cr}$ = 64282 $gmol^{-1}$, with 63 % conversion of AZMA, and DP_{AZMA} = 63.

Catalytic test with **Copo-1-Cr** (2 mol %) 13.4 mg, cyclohexene oxide (30 mg, 0.305 mmol), Et_2O (300 µL), dodecane (20 µL), trimethylsilyl azide (60 µL, 0.458 mmol).
Catalytic test with **Copo-2-Cr** (2 mol %) 7.4 mg, cyclohexene oxide (30 mg, 0.305 mmol), Et_2O (300 µL), dodecane (20 µL), trimethylsilyl azide (60 µL, 0.458 mmol).

Catalytic test with **Copo-3-Cr** (2 mol %) 6.2 mg, cyclohexene oxide (30 mg, 0.305 mmol), Et_2O (300 µL), dodecane (20 µL), trimethylsilyl azide (60 µL, 0.458 mmol).

V. GC traces for the ARO reaction in heterogeneous conditions

GC analyses were performed on a Shimadzu GC 2010 plus using a chiraldex β-PM column (50m × 0.25mm × 0.12µm) and hydrogen as a carrier gas (isothermal 110 °C). Conversion was calculated using dodecane as an internal standard.

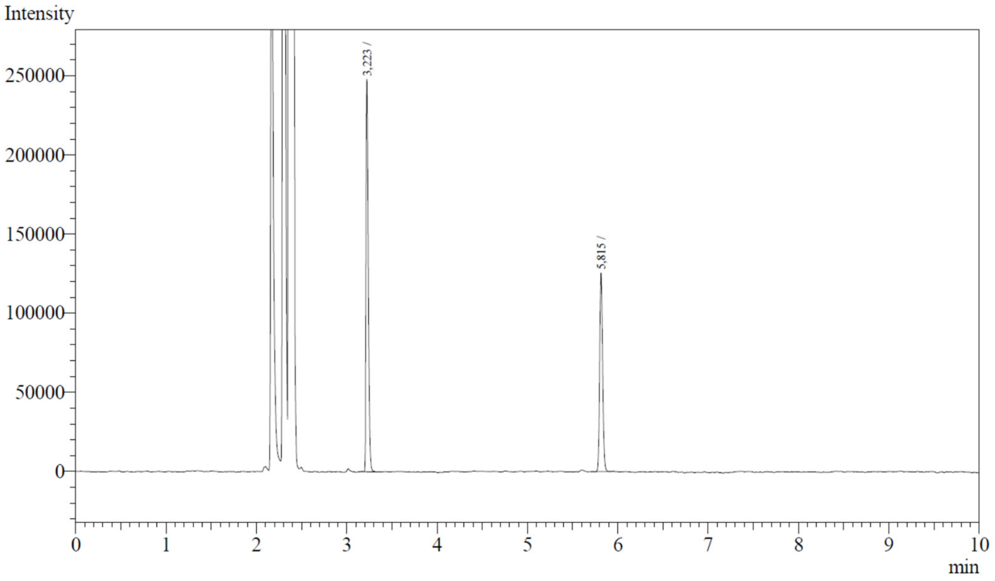

Peak#	Ret.Time	Area	Height	Conc.	Unit	Mark	ID#	Cmpd Name
1	3,223	440076	246259	60,806				
2	5,815	283665	124338	39,194				
Total		723741	370597					

Figure S8: GC spectrum of cyclohexenoxide (Ret.Time 3.22 min) using dodecane (Ret.Time 5.81 min) as a standard.

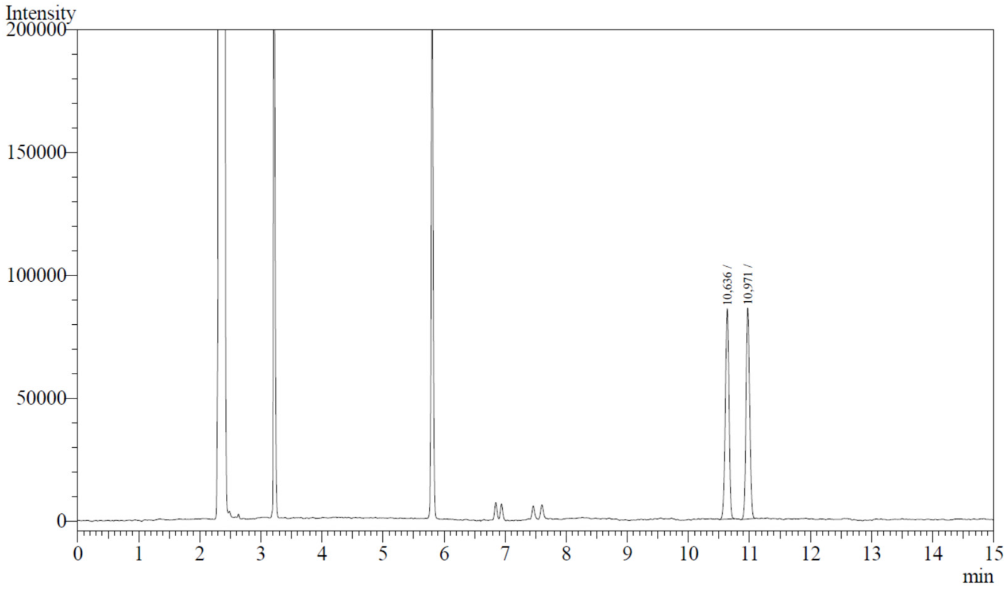

Peak#	Ret.Time	Area	Height	Conc.	Unit	Mark	ID#	Cmpd Name
1	10,636	358588	85392	50,126				
2	10,971	356792	85648	49,874				
Total		715380	171040					

Figure S9: GC spectrum of a racemic sample of ((2-azidocyclohexyl)oxy)trimethylsilane.

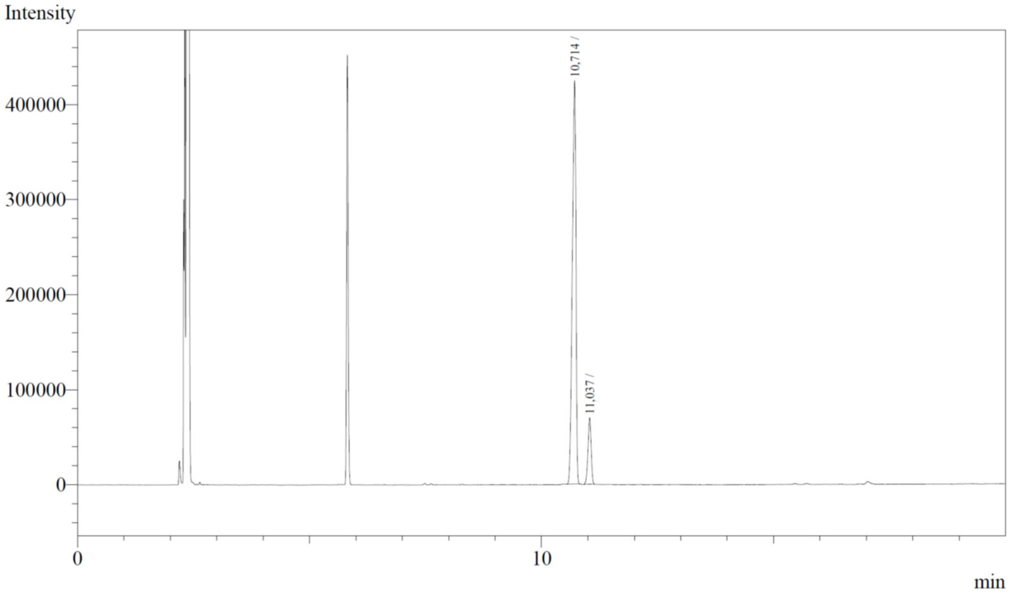

Peak#	Ret.Time	Area	Height	Conc.	Unit	Mark	ID#	Cmpd Name
1	10,714	2297027	424154	87,488				
2	11,037	328498	69795	12,512				
Total		2625525	493949					

Figure S10: GC spectrum of a 75% *ee* sample of ((2-azidocyclohexyl)oxy)trimethylsilane.

VI. ^{1}H and ^{13}C NMR spectra

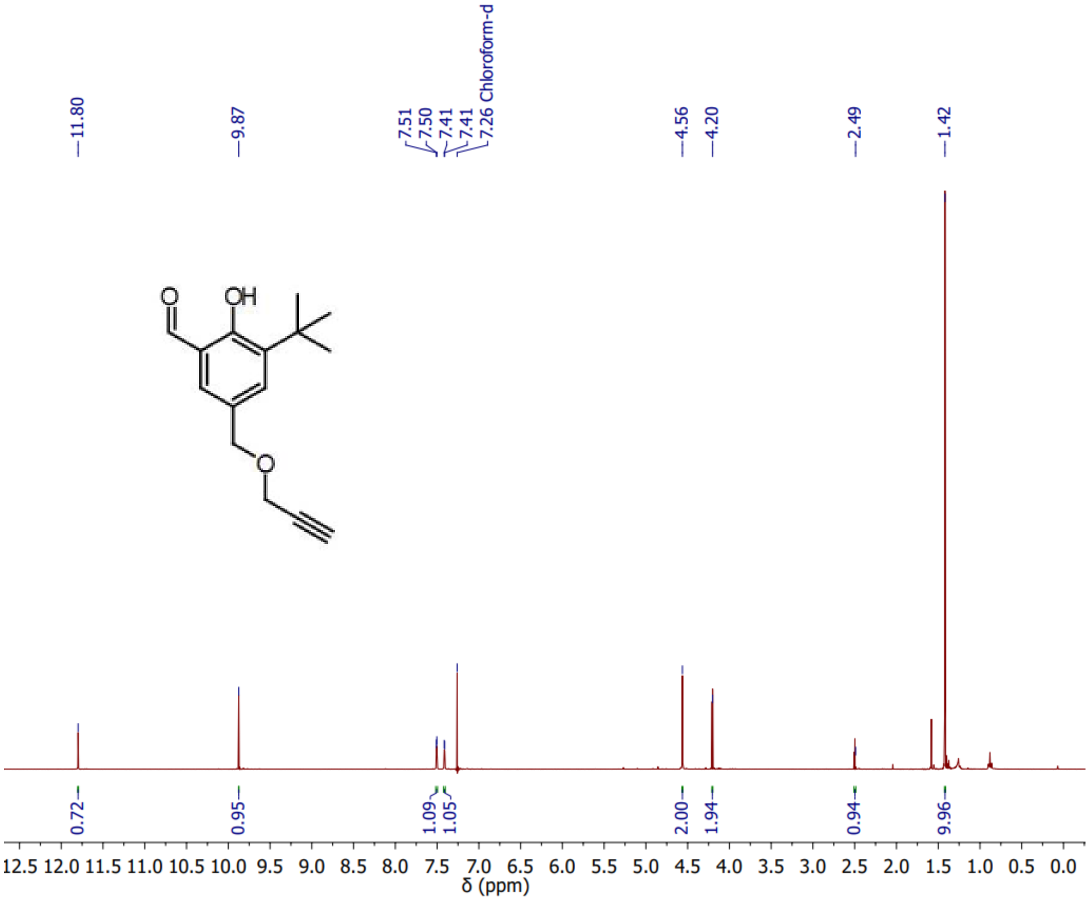

Figure S11: ^{1}H NMR of 3-*tert*-butyl-2-hydroxy-5-((prop-2-ynyloxy)methyl)benzaldehyde.

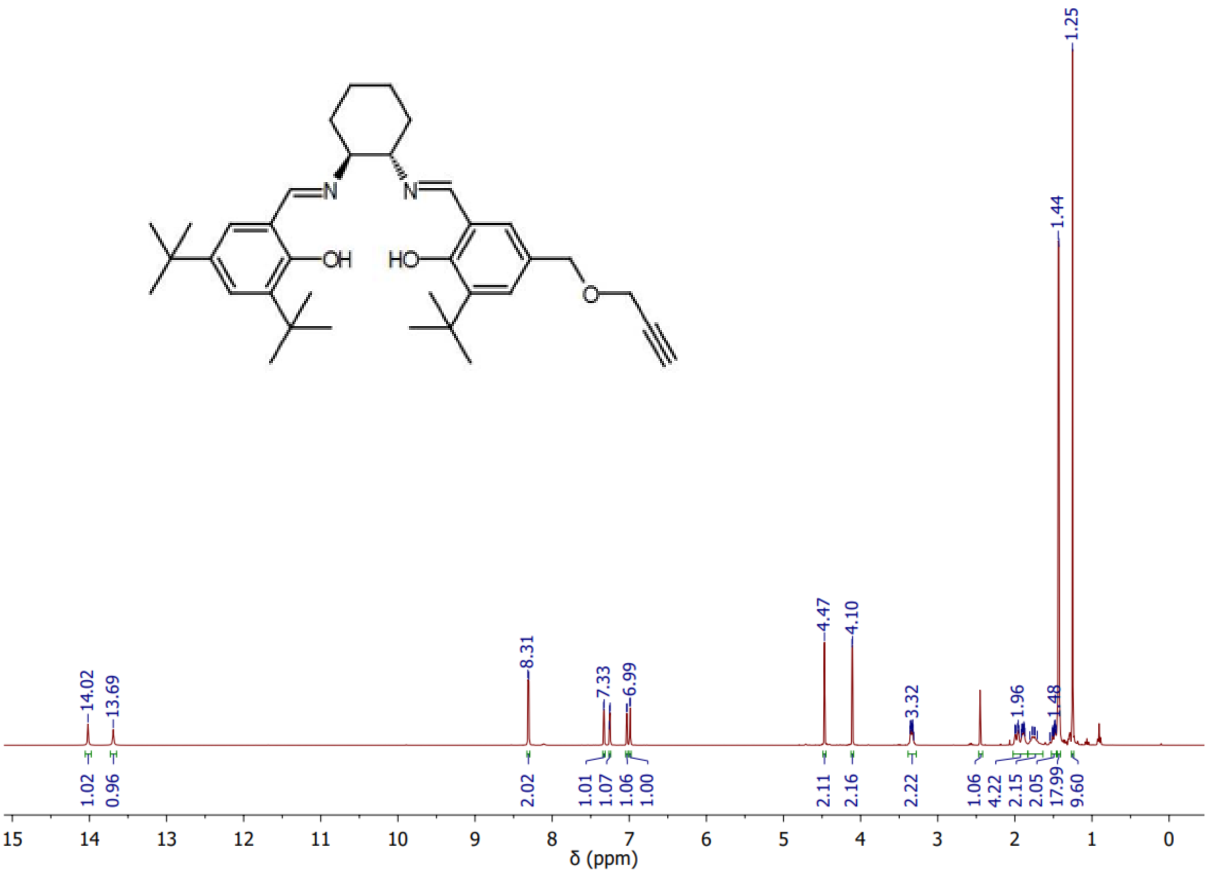

Figure S12: ^{1}H NMR of salen ligand with propargyl moiety.

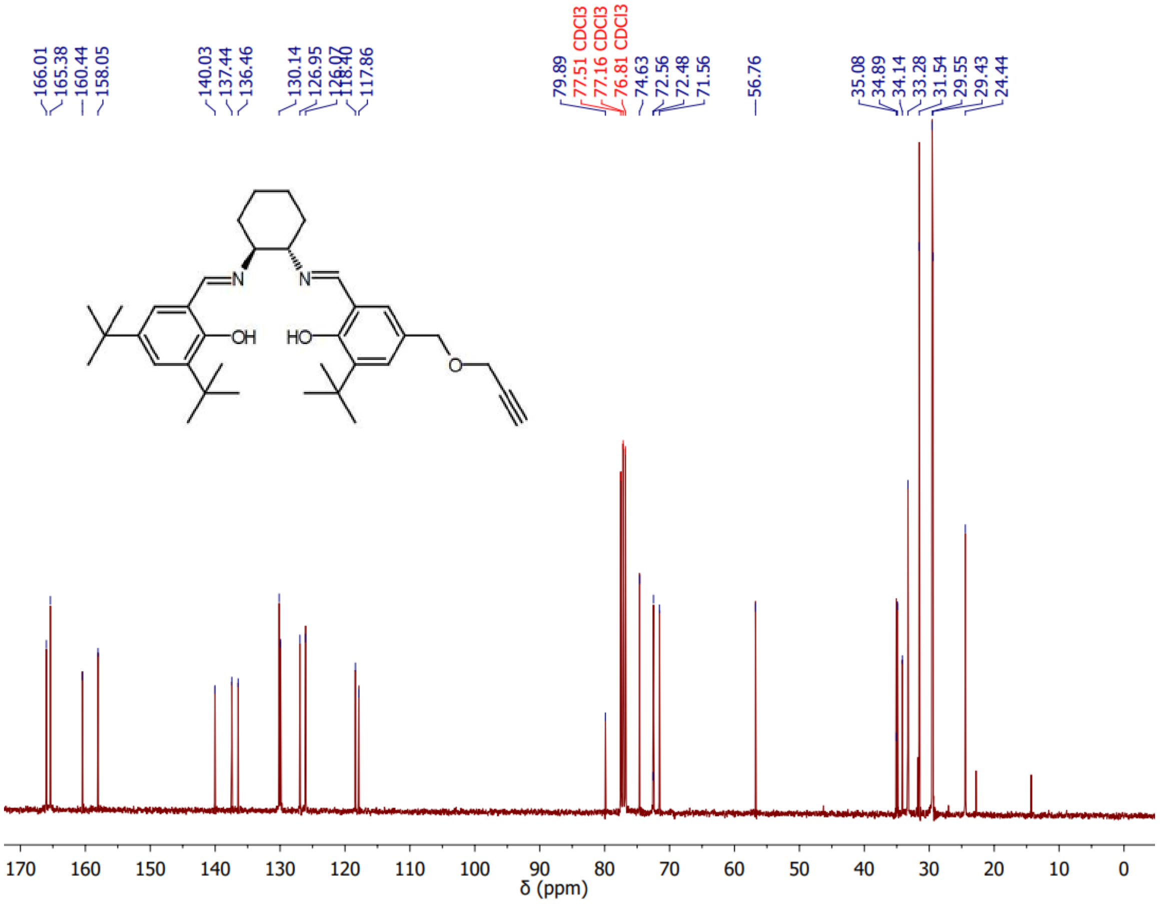

Figure S13: ^{13}C-NMR spectrum of salen ligand with propargyl moiety.
